# High-throughput Gene Tagging in *Trypanosoma brucei*

**DOI:** 10.3791/54342

**Published:** 2016-08-12

**Authors:** Philip Dyer, Samuel Dean, Jack Sunter

**Affiliations:** ^1^Sir William Dunn School of Pathology, University of Oxford

**Keywords:** Immunology, Issue 114, High-throughput, endogenous gene tagging, PCR, *Trypanosoma brucei*, 96-well electroporation, fluorescent protein, localization

## Abstract

Improvements in mass spectrometry, sequencing and bioinformatics have generated large datasets of potentially interesting genes. Tagging these proteins can give insights into their function by determining their localization within the cell and enabling interaction partner identification. We recently published a fast and scalable method to generate *Trypanosoma brucei* cell lines that express a tagged protein from the endogenous locus. The method was based on a plasmid we generated that, when coupled with long primer PCR, can be used to modify a gene to encode a protein tagged at either terminus. This allows the tagging of dozens of trypanosome proteins in parallel, facilitating the large-scale validation of candidate genes of interest. This system can be used to tag proteins for localization (using a fluorescent protein, epitope tag or electron microscopy tag) or biochemistry (using tags for purification, such as the TAP (tandem affinity purification) tag). Here, we describe a protocol to perform the long primer PCR and the electroporation in 96-well plates, with the recovery and selection of transgenic trypanosomes occurring in 24-well plates. With this workflow, hundreds of proteins can be tagged in parallel; this is an order of magnitude improvement to our previous protocol and genome scale tagging is now possible.

**Figure:**
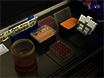


## Introduction

*Trypanosoma brucei* is a protozoan parasite that causes human African trypanosomasis and nagana in cattle. *T. brucei* is an ideal organism for the analysis of protein function due to the combination of a high quality genome, numerous proteomics and transcriptomics datasets and well developed molecular tools ^1-3^. Advances in proteomics and sequencing have resulted in large datasets that highlight potentially interesting genes ^4-6^; however, many genes have minimal information associated with them in the existing databases. There is therefore a need for a high-throughput method to aid protein functional characterization.

Expression of a tagged protein can give a multiplicity of insights into a protein's function. For example, a protein tagged with a fluorescent protein or epitope can be localized by fluorescence microscopy, which gives information about where the protein might be exerting its biological effect. Alternatively, a protein tagged with a TAP^ 7^, HaloTag ^8^ or His tag can be purified for biochemical assays and identification of its interaction partners.

We recently developed a robust tagging methodology for *T. brucei*^  9^. This used long primer PCR to generate the DNA for transfection and allowed the tagging of dozens of proteins in parallel – a major improvement to existing protocols. We have now improved the scalability of this protocol in procyclic forms by an order of magnitude. Here, we present our method where we perform the PCR and transfection into 96-well plates, with the recovery and selection occurring in 24-well plates. As hundreds of proteins can now be tagged in parallel this method provides a cost-effective and feasible method for tagging the entire trypanosome genome.

## Protocol

### 1. 96-well Long Primer PCR

1. Pre-freeze 96-well PCR cooler in -80 °C freezer.
2. In a 10 ml tube, prepare Master Mix A: 2,100 µl ddH_2_O, 105 µl PCR grade DMSO, 105 µl 10 mM dNTPs, 105 µl pPOT template (25 ng/µl) ^9^, for a total volume of 2,415 µl per plate.
3. Aliquot 23 µl into each well of a 96-well PCR plate.
4. Add 2 µl of 100 µM pooled primers to each loaded well using a P20 12 channel multichannel pipette. Seal with film, place plate on the pre-chilled PCR cooler and allow to freeze for a minimum of 20 min at -80 °C.
5. In a 10 ml tube, prepare Master Mix B: 2,048 µl ddH_2_O, 525 µl 10x buffer, 52.5 µl polymerase for a total volume of 2,626 µl per plate.
6. Remove plate and PCR cooler from the -80 °C freezer.
7. With the plate still on the PCR cooler, add 25 µl Master Mix B on top of the frozen Master Mix A for a total reaction volume of 50 µl. This is time critical, and should be completed before Mix A can thaw. Seal plate with film.
8. Set thermal cycler as follows: 94 °C for 10 min, then 30 cycles of 94 °C for 15 sec, 65 °C for 30 sec, 72°C for 2 min followed by a final extension period of 72 °C for 7 min.
9. Load the PCR plate after the block has reached 94 °C.

### 2. Validation of 96-well Long Primer PCR

1. Cast a large 1% (w/v) agarose gel in Tris Acetate EDTA (TAE) running buffer with 4 x 28-well combs to give a gel with 4 rows of wells. Align alternate teeth of the comb with the tips of a 12 channel multichannel pipette. Carefully submerge the gel in TAE running buffer after the lanes are loaded.
2. Load 1 kb DNA ladder into the 1^st^ and 28^th^ well of each row.
3. Using a P20 12 channel multichannel pipette, transfer 2 µl of PCR product directly to each lane of the gel (**Figure 1A**). Do this quickly to reduce the possibility of contaminating the amplicons.
  1. Load the PCR products from row A of the PCR plate into the odd numbered lanes of the 1st row of the gel (A1 - lane 3, A2 - lane 5 …… A11 - lane 23, A12 - lane 25) and load the PCR products from row B of the PCR plate into the even numbered lanes of the 1^st^ row of the gel (B1 - lane 4, A2 - lane 6 …… B11 - lane 24, B12 - lane 26).
  2. Load the PCR products from row C and D of the PCR plate using the same pattern as described above, with row C into the odd numbered lanes and row D into the even numbered lanes. Continue this loading pattern until each well of the PCR plate is loaded onto the gel. DNA loading buffer is not required to load this gel.
4. Place the gel into the running tank and fill the tank with TAE running buffer until the gel is submerged. Run at 100 V for 30 min and visualize using a UV transilluminator. See **Figure 1B** for an example gel. Note: PCR products can be stored at -20 °C for several weeks prior to transfection

### 3. 96-well Transfection

1. Use the *T. brucei* procyclic form cell line SMOXP9 ^10^ for this procedure and grow in SDM-79 media containing 10% FCS ^10^.
2. Use 1 x 10^7^ cells per transfection (total of 1.1 x 10^9^ cells) when tagging on the N terminus of the protein and 2 x 10^7^ cells per transfection (total of 2.2 x 10^9^ cells) when tagging on the C terminus of the protein. Maintain the cells in mid-log (1.2 x 10^6^-1 x 10^7^ cells/ml) for several days prior to transfection and harvest the cells for transfection at a density of 5-8 x 10^6^ cells/ml.
3. Allow frozen PCR products for transfection to thaw at room temperature.
4. Count cells using a haemocytometer or automatic cell counter.
5. Pellet required number of cells in multiple 50 ml tubes at 800 x g for 10 min.
6. After centrifugation discard supernatant and resuspend the cells in 10 ml modified cytomix (0.8 mM EGTA, 24 mM KCl, 0.15 mM CaCl_2_, 10 mM potassium phosphate buffer pH7.6, 25 mM HEPES-KOH pH7.6, 2.6 mM MgCl_2_, 0.5% (w/v) glucose, 100 µg/ml BSA, 1 mM hypoxanthine, 144 mM sucrose) per tube. Transfer all cell solutions to a single tube and spin again at 800 x g for 10 min.
7. After centrifugation, discard supernatant and resuspend the cells in 23 ml of modified cytomix for a final concentration of 5 x 10^7^ cells/ml.
8. While cells are spinning, add 1 ml of SDM-79 media to each well of 4 x 24-well tissue culture plates. Label the plates A-D and draw a ring around well A1 on each plate in permanent marker to help plate orientation.
9. Connect the plate handler to the electroporator. On the electroporator unit set the voltage to 1,500 V, the pulse length to 100 µsec and the number of pulses to 12 and the pulse interval to 500 msec. On the plate handler, set the pulse count to 1. This ensures that that electroporator will apply a single pulse to each of the 12 columns of the electroporation plate.
10. Using a P200 12 channel multichannel pipette, transfer the PCR reactions from the PCR plate to the 96-well disposable electroporation plates, 4 mm gap.
11. When the cells have been spun and are resuspended to the final concentration of 5 x 10^7^ cells/ml (Step 3.7), transfer to a reagent reservoir. Pipette 200 µl of the cell solution into each well of the electroporation plate using a P200 12 channel multichannel pipette, mix with PCR product by pipetting.
12. Using tissue remove any droplets from the top of the plate to avoid short-circuits.
13. Apply the sealing film provided in the plate packaging to the top of the electroporation plate, positioning it so as to leave the holes for the electrodes uncovered at the top and bottom of each column. Avoid covering the raised points used to guide the film.
14. Load the electroporation plate into the plate handler and close the lid. Press 'Pulse' on the electroporator unit.
15. After electroporation, quickly transfer the cells from the 96-well electroporation plate to the 4 x 24-well tissue culture plates. To transfer the cells, use a P200 12 channel multichannel pipette with every other tip missing such that the remaining 6 tips line up with the 6 rows on a 24-well tissue culture plate. Transfer the electroporated cells in the 96-well electroporations plates in pre-defined pattern to the 24-well tissue culture plates (**Figure 2A**).
16. Prepare the P200 pipette tips - for each 96-well transfection prepare 2 boxes of 96 tips with every other column removed.
  1. Transfer wells A1/A3/A5/A7/A9/A11 on the 96-well electroporation plate to plate A row A of the 24-well tissue culture plates, B1/B3/B5/B7/B9/B11 into row B, C1/C3/C5/C7/C9/C11 into row C and D1/D3/D5/D7/D9/D11 into row D. After transfer of each set of 6 wells from the 96-well plate to the 24-well plate, the wells in the 96-well plate are then washed with media from the corresponding wells in the 24-well plate and the wash is then transferred back to the wells in the 24-well plate.
  2. Transfer wells E1/E3/E5/E7/E9/E11 on the 96-well electroporation plate to 24-well plate B row A, F1/F3/F5/F7/F9/F11 into row B, G1/G3/G5/G7/G9/G11 into row C and H1/H3/H5/H7/H9/H11 into row D. After transfer of each set of 6 wells from the 96-well plate to the 24-well plate, the wells in the 96-well plate are then washed with media from the corresponding wells in the 24-well plate and the wash is then transferred back to the wells in the 24-well plate.
  3. Transfer wells A2/A4/A6/A8/A10/A12 on the 96-well electroporation plate to 24-well plate C row A, B2/B4/B6/B8/B10/B12 into row B, C2/C4/C6/C8/C10/C12 into row C and D2/D4/D6/D8/D10/D12 into row D. After transfer of each set of 6 wells from the 96-well plate to the 24-well plate, the wells in the 96-well plate are then washed with media from the corresponding wells in the 24-well plate and the wash is then transferred back to the wells in the 24-well plate.
  4. Transfer wells E2/E4/E6/E8/E10/E12 on the 96-well electroporation plate to 24-well plate D row A, F2/F4/F6/F8/F10/F12 into row B, G2/G4/G6/G8/G10/G12 into row C and H2/H4/H6/H8/H10/H12 into row D. After transfer of each set of 6 wells from the 96-well plate to the 24-well plate, the wells in the 96-well plate are then washed with media from the corresponding wells in the 24-well plate and the wash is then transferred back to the wells in the 24-well plate.
17. Using an inverted phase contrast microscope, examine 1 well from each column to check the cells. There will be a mixture of live and dead cells with a substantial amount of cell debris. Live cells can be distinguished from dead cells because they will be bright under phase contrast microscopy and will be moving.
18. Place the 24-well plates in a clean, plastic box with a lid that forms a tight seal. Put the box in a 28 °C incubator.
19. Between 6-8 hr later, add 1 ml of SDM-79 with 10% FCS containing 2x selective antibiotic to each well. In our experience, cell lines where the genes are tagged on the N terminus are resistant to higher concentrations of selective drug. Therefore, using blasticidin as an example, tagging on the N terminus requires a final concentration of 20 µg/ml blasticidin and tagging on the C terminus requires a final concentration of 10 µg/ml blasticidin. Note: Transgenic cell lines will become visible 9-10 days after transfection.
20. Passage at least twice in fresh drug and media prior to analysis. **Figure 2B **shows the results from a typical 96-well transfection for tagging 96 different proteins on the N terminus. Assess each well by phase contrast microscopy to determine if it contains healthy, drug resistant cells. A healthy well contains predominantly (<95%) highly active cells that are bright in phase contract microscopy. Subsequent analysis by epifluorescence microscopy or western blot is required to determine if the protein has been successfully tagged.

## Representative Results

In this representative transfection, the primers were designed using the TagIt perl script^ 9^ and synthesized commercially. The 96-well PCR was performed and validated as described (**Figure 1A**); in this example, 95/96 PCRs were successful (**Figure 1B**). In our experience, repeating failed reactions either with the original primers or re-synthesized primers does not result in a successful PCR.

The amplicons were transferred into 96-well electroporation plates for electroporation (**Figure 2A**) and then transferred to 24-well culture plates for recovery and selection. After sufficient time to allow the selection to occur, the wells were scored for survival prior to further analysis. In this example, 88/96 wells (92%) were positive after 15 days selection.


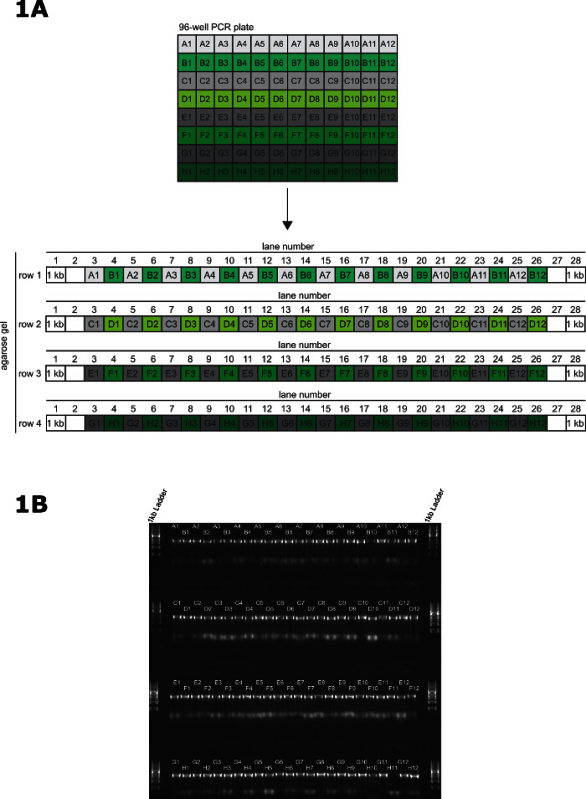
**Figure 1: Validation of long primer PCR. **(**A**) Pattern for transferring PCR products from 96-well PCR plate to the agarose gel for validation of PCR. (**B**) A representative 96-well PCR validation gel for tagging 95 genes on the N terminus, and 1 amplicon containing no 5' targeting homology to act as a negative control in the transfection. The samples are loaded onto a 1% (w/v) agarose gel and resolved at 100 V for 30 min. Please click here to view a larger version of this figure.


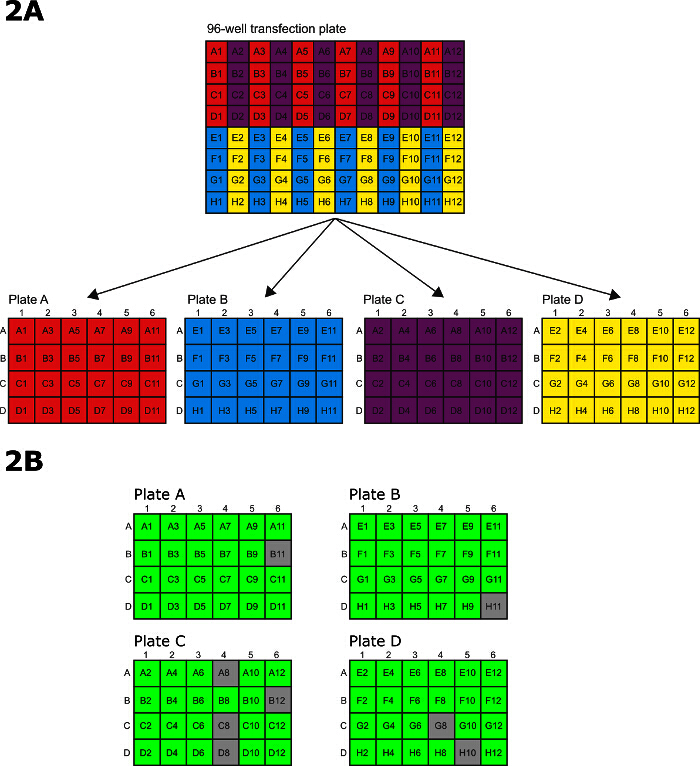
**Figure 2: 96-well electroporation and selection.** (**A**) Pattern for transferring electroporated cells from 96-well electroporation plate to 24-well tissue culture plates. (**B**) A schematic showing a typical live/dead scoring after 15 days of selection in 24-well plates. Green represents a successful transfection, grey represents a well with only dead cells. In this example, 88/96 (92%) of wells contained successfully transfected parasites. Please click here to view a larger version of this figure.

## Discussion

Dramatic improvements in the sensitivity of proteomics and transcriptomics methods in the last 5-10 years has provided valuable data on thousands of genes and their products. However, the tools to address the function of these proteins have not kept pace.

Tagging a protein facilitates numerous experiments to determine its function. For example, a protein can be fused to a fluorescent protein in a variety of different colors to facilitate localization and co-localization studies. Tags developed for electron microscopy, such as APEX2 or miniSOG ^11,12^, allow ultrastructural localization of the tagged protein. Tags for biochemistry, such as the TAP tag, and ProtC-TEV-ProtA (PTP) tag ^7,13^ allow purification of complexes associated with the protein for identification of binding partners or *in vitro* biochemical assays.

The specific steps that are critical to the success of the protocol are: the incorporation of DMSO into the PCR Master Mix 1, freezing of the PCR Master Mix 1 prior to the addition of the Master Mix 2, the use of the commercial polymerase in Master Mix 2 and the modification of the cytomix electroporation buffer. In our experience, it is necessary to use double the number of cells for C terminal tagging transfections as for N terminal tagging transfections in order to achieve a similar proportion of positive wells. Therefore, all steps should be performed as described.

This technique is only likely to be successful when transfecting the insect procyclic form trypanosome. Bloodstream forms have a lower transfection efficiency^ 14^, moreover they are likely to die during selection due to density-dependent toxicity that is unrelated to the selective drug. Therefore, our previous protocol represents the current best technology for tagging of bloodstream form trypanosomes^ 9^. It is also likely that the transfection efficiency will vary dependent upon the specific trypanosome isolate. This protocol was optimized using 927 SMOX procyclic forms – other strains may require additional optimization. Measures that may increase the probability of success include: increasing the amount of PCR amplicon, increasing the number of cells included in the transfection.

We present a method where hundreds of proteins can be tagged in parallel. This will facilitate large scale studies on localization and interaction, complementing existing large datasets and providing invaluable information to the community. Primer templates are available upon request and a list of plasmids templates is available from: http://www.sdeanresearch.com/

## Disclosures

 The authors have nothing to disclose.
